# Risk factors for pancreatic cancer in electronic health records: an umbrella review of systematic reviews and meta-analyses

**DOI:** 10.1016/j.eclinm.2025.103297

**Published:** 2025-06-14

**Authors:** Sarah F. Moore, Sarah Price, Judit Konya, Sophie Blummers, Fiona M. Walter, Richard D. Neal, Gary Abel

**Affiliations:** a Faculty of Health and Life Sciences, University of Exeter, UK; b Wolfson Institute for Population Health, QMUL, UK

**Keywords:** Pancreatic cancer, Risk factors, Umbrella review

## Abstract

**Background:**

Pancreatic cancer has poor survival because of predominantly advanced-stage diagnosis. One strategy for improving outcomes is earlier identification, possibly achievable by enhanced surveillance or improved risk prediction modelling. This umbrella review updates previous evidence with a comprehensive assessment of factors which could inform risk assessments.

**Methods:**

Database searches were performed in MEDLINE and EMBASE via Ovid and the Science Citation Index Expanded of the Web of Science Core collection from inception to March 2025. Systematic reviews and meta-analyses of factors associated with altered risk of pancreatic cancer, available in a coded electronic healthcare record, were included. Participants in component studies were adults, and we compared exposed/not exposed/differentially exposed participants. There was no geographical restriction. The main outcome was potential risk factors for pancreatic cancer, categorised by degree of association. The study was registered with PROSPERO, registration number CRD42024526338.

**Findings:**

2386 abstracts and 449 full texts were dual screened, resulting in 168 studies included in the review, comprising 365 meta-analyses of individual risk factors or strata and >2,255,495 pancreatic cancer cases. 21 meta-analyses reported gender-disaggregated data which were extracted and reported separately.

Of the 80 potential risk factors identified, 38 were associated with an increased pancreatic cancer risk, 11 with a protective effect, and 31 had no significant association with pancreatic cancer. Major newly found risk factors were autoimmune liver disease, BRCA gene mutation, co-infection with hepatitis B and C, and insulin use.

**Interpretation:**

This comprehensive umbrella review of pancreatic cancer risk factors provides an up-to-date summary useful for identifying prevention and surveillance approaches, and for developing risk prediction models and directing future research.

**Funding:**

This work was supported by a 10.13039/100004440Wellcome doctoral fellowship for primary care clinicians for Sarah Moore (PMHG1A4).

**Research in context**

**Evidence before this study**

Our searches across MEDLINE and EMBASE via Ovid and the Science Citation Index Expanded database on the Web of Science Core Collection using the key concepts of ‘pancreatic cancer’, ‘risk factors’, and ‘systematic review’ identified 6 studies that took a broad approach to identifying risk factors for pancreatic cancer. Of these, the study by Maisonneuve and Lowenfels from 2015 followed methods and reported results closest to our study. They identified 39 risk factors, of which 22 matched our criteria. However, this review was from nearly ten years ago and there has since been a substantial increase in the numbers of studies on risk factors for pancreatic cancer.

**Added value of this study**

This umbrella review provides an up-to-date comprehensive summary of evidence on risk factors for pancreatic cancer. When compared to the review of Maisonneuve and Lowenfels from 2015, 52 new risk factors have been identified that have been investigated in systematic reviews or meta-analyses to assess their association with pancreatic cancer, including autoimmune liver disease, BRCA gene mutation, co-infection with hepatitis B and C, and insulin use. We have also changed the risk categorisation of 8 of the 22 originally reported risk factors as a result of updated evidence; for example, increasing the risk estimate in people with a family history of pancreatic cancer.

**Implications of all the available evidence**

This umbrella review provides an up-to-date summary of evidence on risk factors for pancreatic cancer that may be used in three key areas: targeting prevention and surveillance to those with identified risk factors; developing risk prediction models using a comprehensive approach to candidate variable identification; and providing direction for future research.

## Introduction

Comprehensive knowledge of the factors which are associated with altered risk of cancer is essential for three key areas associated with improving outcomes for patients: targeting prevention and surveillance to high risk individuals, developing risk prediction models and providing direction for future research. For disambiguation of terms used in this paper, please see Box 1.

Box 1 **Defining terms: features, factors and variables, taken from Moore et al.1**

In the literature surrounding risk prediction models, several terms are used interchangeably and can cause confusion. We will therefore clarify how we are using each term for the purposes of this paper.

A dataset is made up of information about an individual, e.g., their age, what medications they take or whether they are smokers. Each piece of information is known as a **variable** and some of these will be of potential relevance to the model and others will not. Those that are identified as potentially relevant are known as the **candidate variables**, from which the **final variables** that will form the basis for the model will be chosen using statistical or machine learning techniques.

**Risk factors**, sometimes referred to simply as **factors**, are variables that are associated with cancer development. A classic example is smoking. Risk factors affect the prior odds of an individual developing cancer. In this review we are identifying **risk factors** for pancreatic cancer that can be used as **candidate variables** for a risk prediction model.

**Features** refers to the signs, symptoms or test results that could indicate an undiagnosed cancer is present. These are not being investigated in this paper but will be important for development of models of current undiagnosed cancer risk for symptomatic patients. N.B. The term features is often used in machine leaning literature to refer to variables.

It has been repeatedly shown that diagnosing cancers earlier leads to improved outcomes.^2^ Although screening has a part to play in early diagnosis, there are currently no nationwide screening programmes for pancreatic cancer. However, targeted surveillance is performed in some populations for individuals identified as having a high risk of cancer, available through specific programs such as the EuroPAC trial to which patients from Europe can be referred for assessment and potential ongoing surveillance.^3^ There is also clinical guidance laid out by multiple organisations including the International Cancer of the Pancreas Screening (CAPS) Consortium^4^ and the American Gastroenterological Association.^5^ However, the definition of high risk in all these situations is limited to those patients with a family history of pancreatic cancer, specific known genetic mutations or conditions such as hereditary pancreatitis. If there was more information available on other risk factors, potentially combined into risk prediction models, then this type of targeted surveillance could be further developed. In addition, patients identified as high risk could be targeted for risk factor modification e.g., smoking cessation. These patients could also be key priorities for future targeted surveillance with novel tools such as multi-cancer detection tests (MCDs).^6^

Outside of screening, most remaining cancer diagnoses are made after patients present with symptoms of cancer.^7^ However, many of the symptoms and features of an undiagnosed cancer are common in lots of diseases (e.g., abdominal pain as a symptom of pancreatic cancer) and it is therefore difficult to tell which patients with symptoms need further assessment for cancer and which can safely be reassured or treated for other causes. A key method for identifying which patients with features of undiagnosed cancer would benefit from referral is the use of risk prediction models. These risk prediction models are often developed using large-scale patient data derived from electronic healthcare records. Similar to models predicting high risk of future cancer, the critical step in model development for symptomatic individuals is identifying factors with the potential to impact risk of undiagnosed cancer. These factors will form the candidate variables for model development. There is currently no common comprehensive approach to ascertaining these factors and this umbrella review aims to address this gap for pancreatic cancer.^1^

Pancreatic cancer is increasingly common and has very poor outcomes, often due to being diagnosed at an advanced stage, for example in England over 10,000 patients a year are diagnosed with pancreatic cancer with 80% diagnosed at an advanced stage and less than 10% surviving beyond 5 years.^8^^,^^9^ This makes it a good target for risk prediction models that might be able to identify patients at higher risk of either undiagnosed or future pancreatic cancer by combining multiple factors from their electronic healthcare record.

Given the importance of trying to diagnose patients earlier, many studies have tried to identify factors that are associated with an altered risk of pancreatic cancer. As the number of these original studies is very high and most have been summarised in systematic reviews and meta-analyses, this study will take the form of an umbrella review, investigating only systematic reviews and meta-analyses.

The most recent comparable comprehensive review of meta-analyses and systematic reviews of potential risk factors for pancreatic cancer was published nearly ten years ago by Maisonneuve and Lowenfels and has already been cited over 340 times, suggesting the importance of the subject matter.^10^ More recent studies have had different focusses such as early-onset pancreatic cancer^11^ patients with new-onset diabetes^12^ or dietary risk factors^13^ or different methodology such as systematic reviews with only narrative reporting.^14^ Since 2015 there have been a substantial number of new systematic reviews and meta-analyses exploring risk factors for pancreatic cancer, indicating a need for this timely update to the literature.

The aim of this umbrella review of systematic reviews and meta-analyses is therefore to provide an up-to-date list of potential risk factors for pancreatic cancer, particularly for the development of risk prediction models. Given most risk prediction models are developed using coded electronic healthcare data, the focus will be on factors that might be available in such records, e.g., comorbidities and medications, and not those that are more likely to be found in research cohorts or free-text records or have more recently been investigated such as dietary factors, novel biomarkers or genetic traits. A secondary aim is to provide an example of this practice that might be applied as a key step in the identification of candidate variables during the development of risk prediction models for other diseases.

## Methods

The methods for this umbrella review are described in detail in a published protocol^1^ and registered online with PROSPERO, registration number CRD42024526338. A summary of the methods and any deviations from the published protocol are described below.

### Search strategy and selection criteria

This is an umbrella review of systematic reviews and meta-analyses examining risk factors for pancreatic cancer.

The first criterion for inclusion was that a study needed to be a systematic review or meta-analysis of component studies with suitable epidemiological design. The next criterion was that the risk factors for pancreatic cancer studied needed to be those that might reasonably be available in a coded electronic patient healthcare record. This meant excluding those factors related to diet and lifestyle or genomics and novel biomarkers. A decision was taken to deviate from the original protocol and make an exception to this for BRCA1 and BRCA2 genes and hereditary pancreatitis (the result of a mutation in PRSS1 gene) as these are well known single gene mutations that are very important risk factors for pancreatic cancer and are commonly coded in electronic healthcare records. The cancer of interest was primary carcinoma of the pancreas in adults, those studies exclusively examining neuroendocrine tumours of the pancreas were excluded. There was no restriction on geographical location of studies. We specified that the final review needed to be available in full text in English but component studies could be in any language.

Searches were performed from inception of the databases to 3rd March 2025. The databases searched were MEDLINE and EMBASE via Ovid and the Science Citation Index Expanded database on the Web of Science Core Collection. The Cochrane database has not been included as their focus is on interventional rather than observational studies. The key concepts used were ‘pancreatic cancer’, ‘risk factors’ and ‘systematic review’ and full details of the final expanded search terms can be found in Appendix 1. These searches were supplemented by examining references of included studies and performing forward and backward citation chasing in Scopus as necessary. Given the design of studies being sought (i.e., meta-analyses and systematic reviews) our searches were restricted to peer-reviewed literature.

The searches were performed by one reviewer (SM) and uploaded to Covidence, the software used to handle all screening and extraction.^15^ Titles and abstracts, along with full texts were all dual screened. Throughout the screening process any discrepancies were identified and resolved by discussion between the reviewers.

### Data analysis

The results from selected studies were extracted by one reviewer (SM) and checked by a second (SP, JK or SB), any discrepancies were then rechecked against the original study by SM and discussed with the checking author. The same process was undertaken for assessing risk of bias using the ROBIS (Risk Of Bias in Systematic Reviews) tool.^16^ This tool was chosen as the majority of our included studies were expected to be meta-analyses and it has been shown it performs better for assessing these than AMSTAR-2 the other main tool used in the literature.^17^

Due to the large number of included studies and wealth of data available, we did not contact original study authors for clarification when data that we planned to include in our final results table were missing but nor did we exclude those studies, simply marking that element of the results missing in the original data table. The exception to this was where there was no p-value reported for the results of the meta-analysis or the largest component study. In these cases, a p-value was calculated from the available data using standard methods.^18^

Many studies examined more than one risk factor or subdivided a risk factor into strata such as time since diagnosis or patient sex or gender (as defined in the original study). In these circumstances all available data were extracted and a separate line in the results table included for each factor or stratum.

Strength of evidence in meta-analyses was assessed according to predefined credibility assessment criteria, which took into account the number of cases, the p-value for the primary result, the level of heterogeneity and the p-value of the largest included study (see Appendix 1, Table S1 for details of how this was scored).^1^ For studies with significant results from meta-analysis, the strength of evidence was graded in ascending order: weak, suggestive, highly suggestive, convincing.

### Statistics

The statistical analysis plan is detailed in the accompanying protocol.^1^ Quantitative synthesis was not performed. The main reported outcome from included meta-analyses was the effect size, a measure of the strength of the relationship between the risk factor and the development of the disease. Studies used different measures, such as relative risk (RR), odds ratio (OR), hazard ratio (HR) or incidence rate ratios (IRR). To describe this effect size, in accordance with common practice in the literature, we treated these as approximately equal, given the usual event rate for pancreatic cancer is less than 10%.^19^^,^^20^

The main outcome of this umbrella review is a descriptive table containing a set of risk factors for pancreatic cancer according to the degree of association with pancreatic cancer, using previously defined criteria from Maisonneuve and Lowenfels' 2015 review (effect size ≥2.0 = high risk, effect size ≥1.5–<2.0 = moderate risk, effect size >1–<1.5 = low risk, and effect size ≥0.5–<1.0 = low to moderate protection).^10^

There is no consensus in the literature on how to deal with multiple reviews or meta-analyses on the same topic in an umbrella review.^21^^,^^22^ Having reviewed the available options, we decided to take a common pragmatic approach to overlapping studies and selected the single study with the largest, most recent meta-analysis with low risk of bias to represent the risk factor in our final results. This allows for easy comparison with a previous comprehensive summary published in 2015.^10^ Another benefit to this approach is that selecting the single largest, most recent or highest quality meta-analysis or systematic review to represent the relationship between the exposure and outcome is a common approach to dealing with the issue of overlapping component studies in multiple meta-analyses on the same topic.^22^ Other systematic reviews and meta-analyses on the same topic were included in our comprehensive summary table, in our graphical representations of results and described in our narrative results section.

### Ethics

Ethics approval is not required for this umbrella review of published literature.

### Role of the funding source

This work was supported by a doctoral fellowship for primary care clinicians awarded to SM by Wellcome, grant number PMHG1A4. The funder of the study had no role in study design, data collection, data analysis, data interpretation, or writing of the report.

## Results

**Fig. 1:**
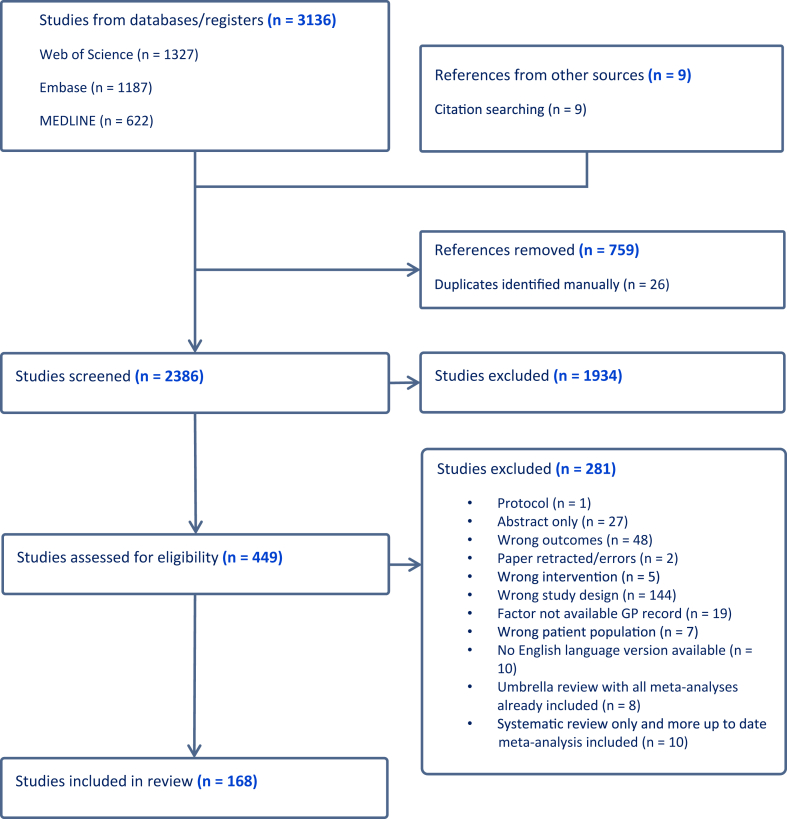
PRISMA flow diagram illustrating study selection.

**Fig. 2:**
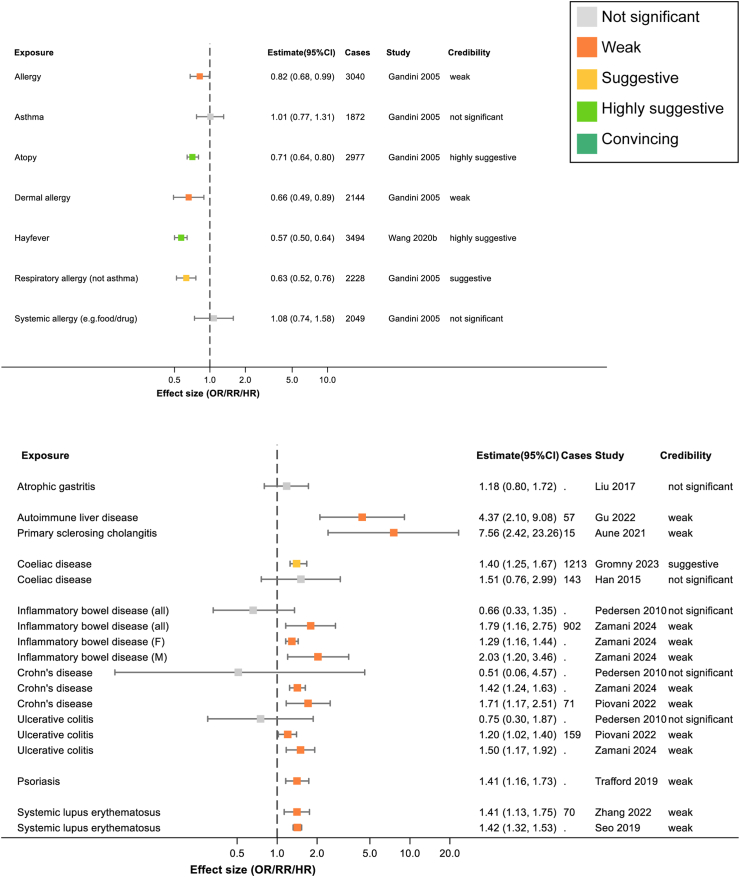
Summary of extracted meta-analyses for allergy (upper panel) and autoimmune disease (lower panel). Results are presented as effect size with 95% confidence intervals. The colours of boxes represent the credibility assessment score for each meta-analysis using criteria described in Methods. Grey: not significant; orange: weak; yellow: suggestive; light green: highly suggestive; dark green: convincing. Cases refers to the number of cases of pancreatic cancer captures in each meta-analysis and full details of each meta-analysis are in Appendix 1, Table S2. OR, odds ratio; RR, risk ratio.

**Fig. 3:**
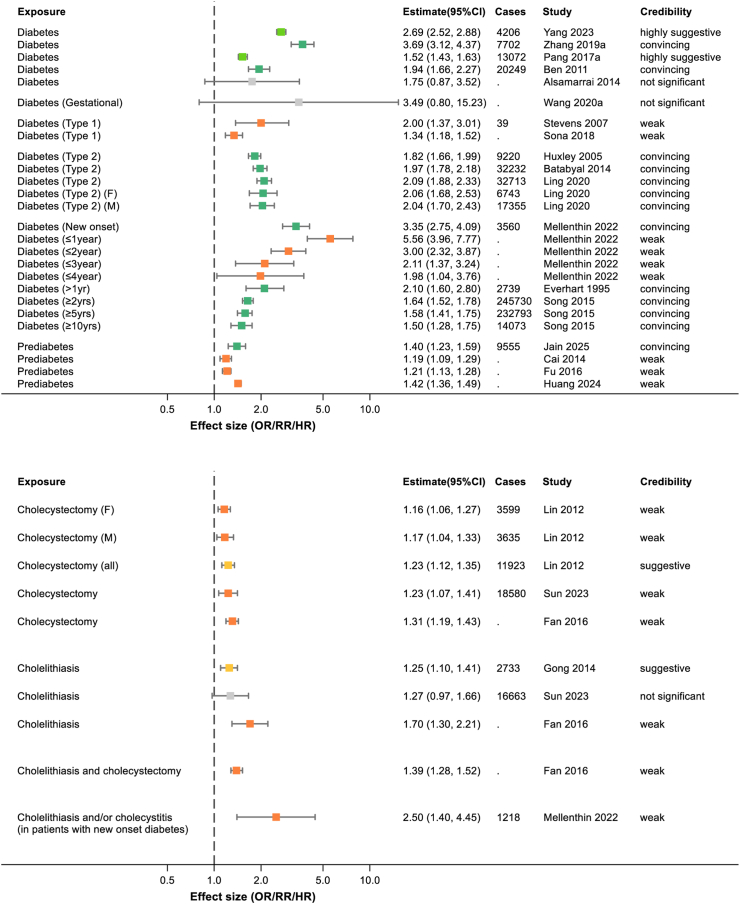
Summary of extracted meta-analyses for diabetes (upper panel) and gall bladder disease (lower panel). Results are presented as effect size with 95% confidence intervals. The colours of boxes represent the credibility assessment score for each meta-analysis using criteria described in Methods. Grey: not significant; orange: weak; yellow: suggestive; light green: highly suggestive; dark green: convincing. Cases refers to the number of cases of pancreatic cancer captures in each meta-analysis and full details of each meta-analysis are in Appendix 1, Table S2. OR, odds ratio; RR, risk ratio.

**Fig. 4:**
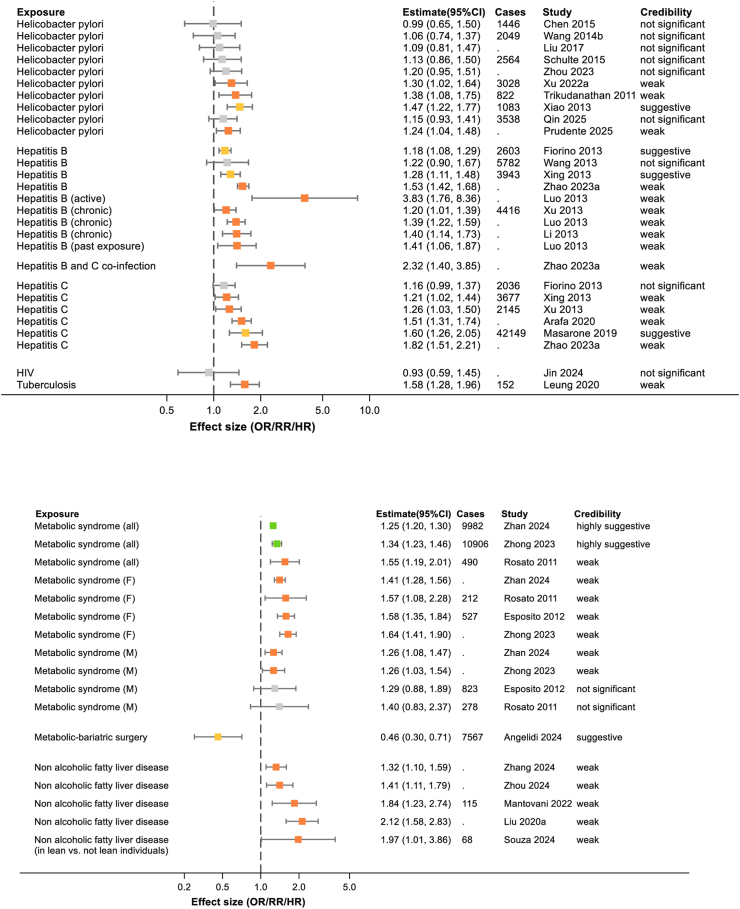
Summary of extracted meta-analyses for infection (upper panel) and metabolic syndrome (lower panel). Results are presented as effect size with 95% confidence intervals. The colours of boxes represent the credibility assessment score for each meta-analysis using criteria described in Methods. Grey: not significant; orange: weak; yellow: suggestive; light green: highly suggestive; dark green: convincing. Cases refers to the number of cases of pancreatic cancer captures in each meta-analysis and full details of each meta-analysis are in Appendix 1, Table S2. OR, odds ratio; RR, risk ratio.

**Fig. 5:**
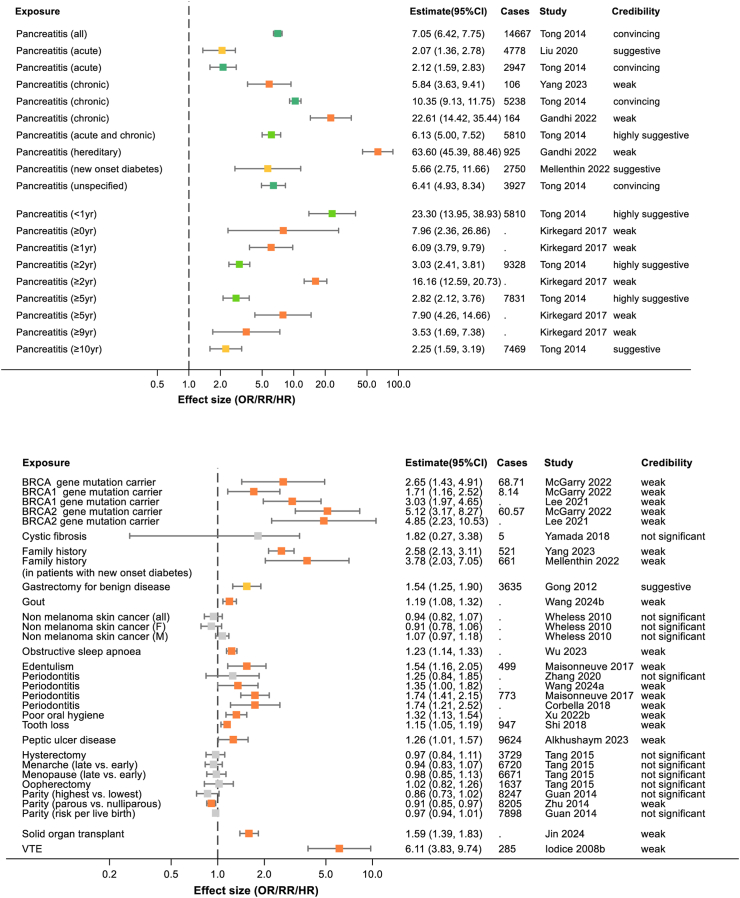
Summary of extracted meta-analyses for pancreatitis (upper panel) and other comorbidities (lower panel). Results are presented as effect size with 95% confidence intervals. The colours of boxes represent the credibility assessment score for each meta-analysis using criteria described in Methods. Grey: not significant; orange: weak; yellow: suggestive; light green: highly suggestive; dark green: convincing. Cases refers to the number of cases of pancreatic cancer captures in each meta-analysis and full details of each meta-analysis are in Appendix 1, Table S2. OR, odds ratio; RR, risk ratio; VTE, venous thromboembolism.

**Fig. 6:**
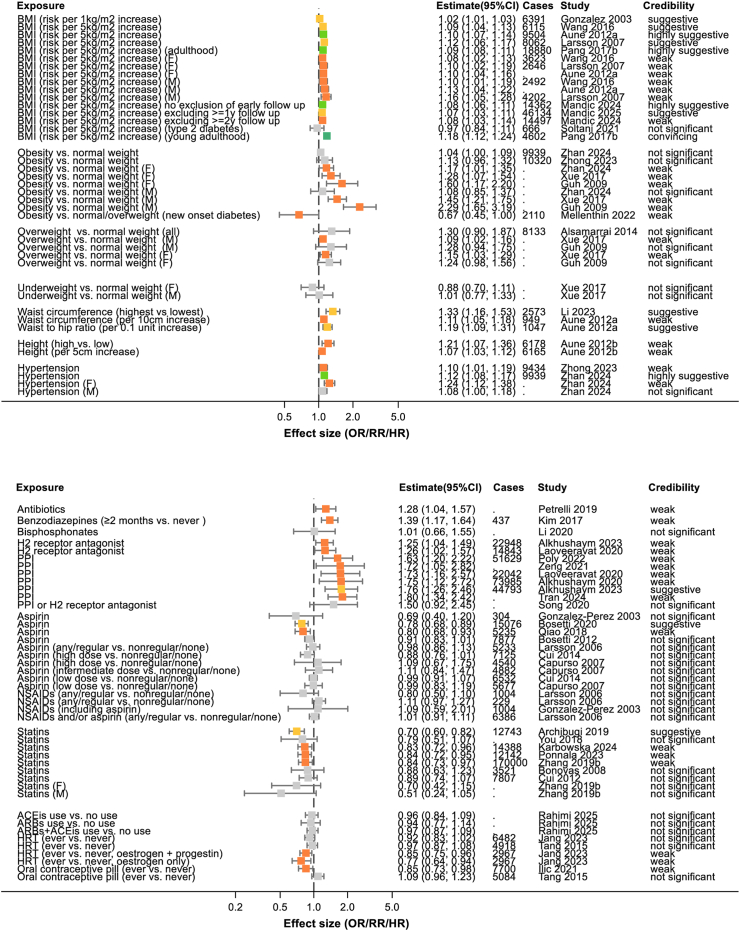
Summary of extracted meta-analyses for anthropometrics (upper panel) and medications (lower panel). Results are presented as effect size with 95% confidence intervals. The colours of boxes represent the credibility assessment score for each meta-analysis using criteria described in Methods. Grey: not significant; orange: weak; yellow: suggestive; light green: highly suggestive; dark green: convincing. Cases refers to the number of cases of pancreatic cancer captures in each meta-analysis and full details of each meta-analysis are in Appendix 1, Table S2. OR, odds ratio; RR, risk ratio; ACEis, angiotensin converting enzyme inhibitors; ARBs, angiotensin receptor blockers; HRT, hormone replacement therapy; NSAIDs, non-steroidal anti-inflammatory drugs; PPI, proton pump inhibitor.

**Fig. 7:**
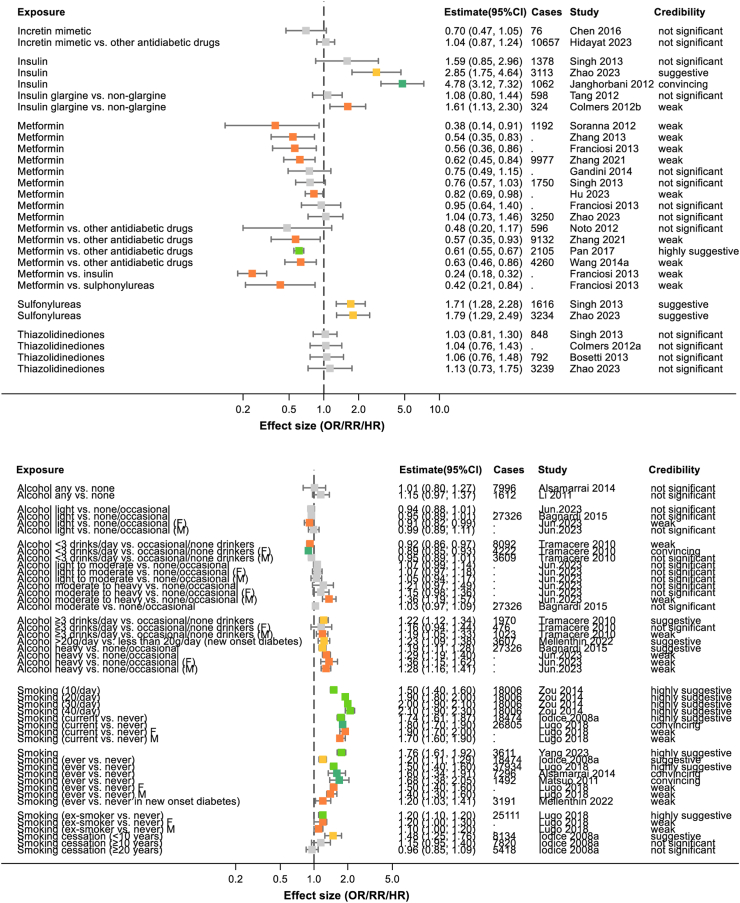
Summary of extracted meta-analyses for diabetic medications (upper panel) and alcohol and smoking (lower panel). Results are presented as effect size with 95% confidence intervals. The colours of boxes represent the credibility assessment score for each meta-analysis using criteria described in Methods. Grey: not significant; orange: weak; yellow: suggestive; light green: highly suggestive; dark green: convincing. Cases refers to the number of cases of pancreatic cancer captures in each meta-analysis and full details of each meta-analysis are in Appendix 1, Table S2. OR, odds ratio; RR, risk ratio.

**Fig. 8:**
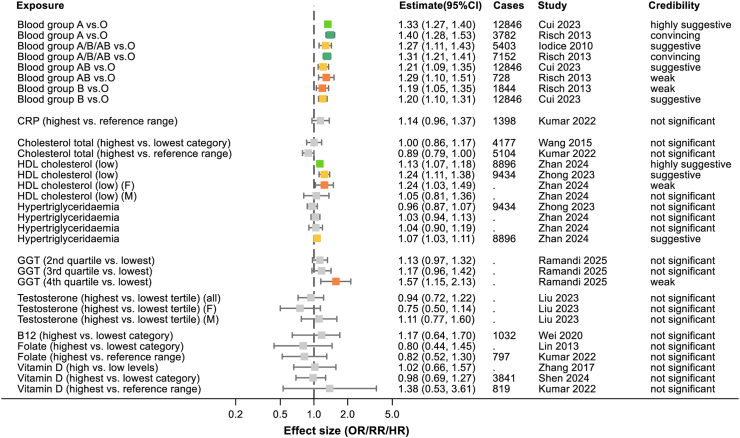
Summary of extracted meta-analyses for test results. Results are presented as effect size with 95% confidence intervals. The colours of boxes represent the credibility assessment score for each meta-analysis using criteria described in Methods. Grey: not significant; orange: weak; yellow: suggestive; light green: highly suggestive; dark green: convincing. Cases refers to the number of cases of pancreatic cancer captures in each meta-analysis and full details of each meta-analysis are in Appendix 1, Table S2. OR, odds ratio; RR, risk ratio; CRP, C-reactive protein; HDL, high-density lipoprotein; GGT, gamma-glutamyl transferase.

2386 abstracts and 449 full texts were dual screened, resulting in 168 studies being included in the review (see Fig. 1 and Appendix 1, Table S3). Of the 168 included studies, there were 6 which only conducted systematic reviews alone. The remaining studies all conducted meta-analyses and, in many cases, multiple meta-analyses of different risk factors or different strata within those risk factors. This resulted in 365 individual meta-analyses being extracted. 230 of these meta-analyses reported numbers of cases of pancreatic cancer in at least some of their included studies and these combined to more than 2,255,495 cases of pancreatic cancer studies across the umbrella review (acknowledging that many of these cases will have appeared in multiple original studies). Where possible data were extracted by sex or gender and these meta-analyses are included in Appendix 1, Table S2 and Fig. 2, Fig. 3, Fig. 4, Fig. 5, Fig. 6, Fig. 7, Fig. 8.

The main results for the included studies are shown in Table 1, which stratifies factors by their degree of association with pancreatic cancer according to the results of the most representative meta-analysis, as per methods (Appendix 1, Table S1). The degree of evidence is described by the credibility assessment score of the same study. It is important to note that these criteria were pre-defined according to the literature and a study could not score higher than ‘weak’ unless it had a report of case numbers, and that number was greater than 1000.^1^ Full results are shown in Appendix 1, Table S2. Results for all meta-analyses are also described narratively by category of risk factor below, complemented by Fig. 2, Fig. 3, Fig. 4, Fig. 5, Fig. 6, Fig. 7, Fig. 8.

**Table 1:** Summary of the associations between risk factors and pancreatic cancer reported in 365 meta-analyses across 163 publications.

Degree of association	Risk factor	Number of studies	Number of studies showing:			Degree of evidence		Notes	Comparison of category with Maisonneuve and Lowenfels 2015^10^
			Inverse association	Null association	Positive association	Credibility assessment	Confirmed in >1 study		
High risk (RR ≥ 2.0)	**Comorbidities**								
	Autoimmune liver diseases	2	0	0	2	+			New
	BRCA gene mutation	2	0	0	2	+	Yes		New
	Family history of pancreatic cancer	1	0	0	1	+		Relationship not specified	Increased
	Hepatitis B and C co-infection	1	0	0	1	+			New
	History of venous thromboembolism^a^	1	0	0	1	+			Same
	Pancreatitis	5	0	0	5	++++	Yes	Acute, chronic or hereditary	Same
	**Medications**								
	Insulin	3	0	1	2	++	Yes	Vs. no insulin in diabetics	New
Moderate risk (RR ≥1.5–<2.0)	**Comorbidities**								
	Diabetes	15	0	1	14	++++	Yes	Any duration since diagnosis and type 2 or type 1, also hyperglycaemia	Same
	Gastrectomy	1	0	0	1	++		For benign disease	Increased
	Hepatitis B infection	7	0	1	6	+	Yes		Increased
	Hepatitis C infection	5	0	1	4	+	Yes		Increased
	Inflammatory bowel disease	2	0	0	2	+	Yes	Including Crohn's disease and ulcerative colitis separately	New
	Solid organ transplant	1	0	0	1	+			New
	Tuberculosis	1	0	0	1	+			New
	**Medications**								
	Proton pump inhibitors	6	0	0	6	+	Yes		New
	Sulfonylureas	2	0	0	2	++	Yes	In diabetics	New
	**Tests**								
	Serum Gamma-Glutamyl Transferase	1	0	0	1	+		Highest vs. lowest quartile	New
	**Other**								
	Smoking	6	0	0	6	++++	Yes	Current vs. never and ever vs. never smoker categories are RR > 1.5 but ex-smoker vs. never risk is lower, RR 1.20	Same
Low risk (RR >1.0–<1.5)	**Comorbidities**								
	Cholecystectomy	3	0	0	3	+	Yes		Same
	Coeliac disease	2	0	1	1	++			New
	Gout^a^	1	0	0	1	+			New
	Hypertension	2	0	0	2	+++	Yes		New
	Metabolic syndrome	4	0	0	4	+++	Yes		Decreased
	Non-alcoholic fatty liver disease	5	0	0	5	+	Yes	One analysis included studies with lean individuals only	New
	Obstructive sleep apnoea	1	0	0	1	+			New
	Peptic ulcer disease^a^	1	0	0	1	+			New
	Prediabetes	3	0	0	3	++++	Yes		New
	Systemic lupus erythematosus	2	0	0	2	+	Yes		New
	**Medications**								
	Antibiotics^a^	1	0	0	1	+			New
	Benzodiazepines	1	0	0	1	+		≥2 months vs. never	New
	H2 receptor antagonists	2	0	0	2	+	Yes		New
	**Tests**								
	Blood group	3	3	0	0	+++	Yes	A/B/AB vs. O	Same
	High density lipoprotein cholesterol (low)	2	0	0	2	++	Yes		New
	Hyperglycaemia	4	0	0	4	+++	Yes		New
	**Other**								
	Alcohol (heavy drinking)	3	0	0	3	++		Heavy vs. none/occasional	Same
	Body mass index (risk per 5 kg/m^2^ increase)	5	0	0	5	+++	Yes	No exclusion of early follow up	New
	Height	1	0	0	1	+			Same
	Waist circumference	2	0	0	2	++	Yes		Same
No association (CI for RR crosses 1.0)	**Comorbidities**								
	Asthma	1	0	1	0	NS			New
	Atrophic gastritis	1	0	1	0	NS			New
	Cholelithiasis	3	0	1	2	NS			New
	Cystic fibrosis	1	0	1	0	NS			New
	Gestational diabetes	1	0	1	0	NS			New
	*Helicobacter pylori* infection	10	0	6	4	NS			Decreased
	Human immunodeficiency virus	1	0	1	0	NS			New
	Hysterectomy	1	0	1	0	NS			New
	Menarche	1	0	1	0	NS		Age at onset late vs. early	New
	Menopause	1	0	1	0	NS		Age at onset late vs. early	New
	Non melanoma skin cancer	1	0	1	0	NS			New
	Oopherectomy	1	0	1	0	NS			New
	Periodontal disease	5	0	2	3	NS		Including periodontitis and poor oral hygiene	New
	Systemic allergy	1	0	1	0	NS			New
	**Medications**								
	Bisphosphonates	1	0	1	0	NS			New
	Hormone replacement therapy	2	0	2	0	NS		Ever vs. never	New
	Incretin-based therapies	2	0	2	0	NS		In diabetics	New
	Non-steroidal anti-inflammatory drugs	2	0	2	0	NS			Same
	Renin-angiotensin-aldosterone system inhibitors	1	0	1	0	NS		ACE inhibitors, angiotensin receptor blockers and combinations	New
	Thiazolidinediones	4	0	4	0	NS		In diabetics	New
	**Tests**								
	B12 serum level	1	0	1	0	NS			New
	Cholesterol level (total)	2	0	2	0	NS			New
	C-peptide	1	0	1	0	NS			New
	C-reactive protein serum level	1	0	1	0	NS			New
	Folate level	2	0	2	0	NS			New
	Hypertriglyceridaemia	2	0	1	1	NS			New
	Testosterone level	1	0	1	0	NS			New
	Vitamin D level	2	0	2	0	NS			Same
	**Other**								
	Alcohol (moderate/low intake vs. no/occasional)	5	1	4	0	NS			New
	Obesity	4	0	2	2	NS			Decreased
	Overweight vs. normal weight	3	0	2	1	NS			New
Low to moderate protection (RR ≥0.5–<1.0)	**Comorbidities**								
	Allergy	1	1	0	0	+			Same
	Atopy	1	1	0	0	+++			New
	Dermal allergy	1	1	0	0	+			New
	Hay fever^a^	1	1	0	0	+++			New
	Metabolic bariatric surgery	1	1	0	0	++			New
	Parity	1	1	0	0	+		Parous vs. nulliparous	New
	Respiratory allergy	1	1	0	0	++		Excluding asthma	New
	**Medications**								
	Aspirin	7	2	5	0	++	Yes		Decreased
	Metformin	10	7	3	0	+	Yes	In diabetics	Same
	Oral contraceptive pill	2	1	1	0	+		Ever vs. never	New
	Statins	9	4	5	0	+	Yes		Decreased

Risk factors are allocated to the relative risk (RR) band of their chosen representative study, explanation of this selection process is detailed in the Methods section. Other included studies may not fall into the same risk category and full details of all studies are shown in Fig. 2, Fig. 3, Fig. 4, Fig. 5, Fig. 6, Fig. 7, Fig. 8 and Appendix 1, Table S2.

Credibility assessment of the evidence in the selected representative meta-analysis: NS = not significant, + = weakly suggestive, ++ = suggestive, +++ = highly suggestive, ++++ = convincing.

a These risk factors only have evidence that was rated as having high or uncertain risk of bias according to ROBIS tool.

### Comorbidities

#### Allergy

Three studies examined the association between allergy and pancreatic cancer, see Fig. 2. The most comprehensive study was also the only one rated as having low risk of bias.^23^ Meta-analysis showed an overall protective effect of having a history of allergy of RR 0.82 (95% confidence interval (95% CI) 0.68, 0.99), which increased when restricted to the studies that adjusted for smoking (RR 0.75; 95% CI 0.65–0.87).^23^ A breakdown of the effects of subtypes of allergy in meta-analyses can be seen in Fig. 2, where atopy (RR 0.71; 95% CI 0.64–0.80)^23^ and hay fever (RR 0.57; 95% CI 0.50–0.64)^24^ show the most credible protective associations.

#### Autoimmune disease

Most autoimmune diseases studied appear to be associated with increased risk of pancreatic cancer, as shown in Fig. 2.

A combined meta-analysis of autoimmune liver diseases, including primary biliary cholangitis, autoimmune hepatitis, and primary sclerosing cholangitis (RR 4.37; 95% CI 2.10–9.08),^25^ and a specific meta-analysis on primary sclerosing cholangitis (RR 7.56; 95% CI 2.42–23.26)^26^ both showed a strong association with pancreatic cancer.

Inflammatory bowel disease was found to have a moderate association with increased pancreatic cancer risk (RR 1.79; 95% CI 1.16–2.75).^27^ Zamani et al. also reviewed two Mendelian Randomisation studies which further underlined this association as they demonstrated ‘genetic liability to inflammatory bowel disease was associated with an increased risk of pancreatic cancer’.^27^

Although coeliac disease was shown to be associated with increased pancreatic cancer risk (RR 1.40; 95% CI 1.25–1.67), the authors of that study counselled significant caution in interpretation of their results and we rated the risk of bias in the study as high because the small number of included studies precluded subgroup analyses.^28^

Systemic lupus erythematosus was consistently shown to have a low-risk association with pancreatic cancer in two meta-analyses with RR 1.41 (95% CI 1.13–1.75)^29^ and RR 1.42 (95% CI 1.32–1.53).^30^ Patients with psoriasis had a very similar magnitude of positive association in a single meta-analysis (RR 1.41; 95% CI 1.16–1.73).^31^

#### Diabetes

Diabetes is clearly associated with an increased risk of pancreatic cancer across 15 meta-analyses (Fig. 3). The risk is higher in new-onset diabetes (RR 3.35; 95% CI 2.75–4.09)^12^ than diabetes of a longer duration (e.g., a diabetes for ≥10 years RR 1.50; 95% CI 1.28–1.75)^32^ and more studies provide evidence for an effect in type 2 diabetes (RR 1.97; 95% CI 1.78–2.18)^33^ than in type 1 diabetes (RR 2.00; 95% CI 1.37–3.01).^34^ However, the increased risk is present even in prediabetes (RR 1.40; 95% CI 1.23–1.59),^35^ a finding that went along with that of an increasing risk of pancreatic cancer (RR 1.14; 95% CI 1.06–1.24) in a linear dose-response for each 10 mg/dl (0.56 mmol/l) increase in fasting glucose, even below the level of diabetes diagnosis.^36^

#### Gallbladder related disease

Although there were several meta-analyses on cholelithiasis that showed positive associations with pancreatic cancer, the largest and most recent study by Sun et al. showed a non-significant effect (RR 1.27; 95% CI 0.97–1.66) overall, though a positive effect was seen in sub-analysis of Asian populations (RR 2.49; 95% CI 1.24–5.02).^37^ The same study did however concur with other meta-analyses in finding a low-risk association between cholecystectomy and pancreatic cancer (RR 1.23; 95% CI 1.07–1.41),^37^ see Fig. 3.

#### Infection

*Helicobacter pylori* infection has been explored extensively for association with pancreatic cancer, with ten meta-analyses included in this review, see Fig. 4. The largest and most recent of these included 17 studies and showed a no association with pancreatic cancer (RR 1.15; 95% CI 0.93–1.41).^38^ Another aspect of *H. pylori* infection that has been investigated is the association with seropositivity of cytotoxin-associated gene A (CagA) and the same study showed no significant association between this variant and pancreatic cancer.^38^

The other extensively investigated association is that between hepatitis infection and pancreatic cancer, specifically hepatitis B and C, see Fig. 4. Both hepatitis B (RR 1.53; 95% CI 1.42–1.68) and hepatitis C (RR 1.82; 95% CI 1.51–2.21) were shown in meta-analyses to have a moderate-risk association with pancreatic cancer across multiple studies, and this association became high-risk in patients co-infected with hepatitis B and C (RR 2.32; 95% CI 1.40–3.85).^39^

Tuberculosis showed a moderate association, with increased risk of pancreatic cancer in a single meta-analysis of three studies (RR 1.58; 95% CI 1.28–1.96).^40^

#### Metabolic syndrome

Metabolic syndrome was shown in two recent studies, each meta-analysing data from around 10,000 patients, to be associated with a low increased pancreatic cancer risk (RR 1.25; 95% CI 1.20–1.30^41^ and RR 1.34; 95% CI 1.23–1.46^42^). In both meta-analyses, this risk appeared to be higher in women with metabolic syndrome than in men with evidence of heterogeneity between groups, for example p = 0.041 in Zhong et al.^42^ Components of metabolic syndrome are described separately in other sections of the results.

Non-alcoholic fatty liver disease (NAFLD), also known as metabolic dysfunction associated steatotic liver disease (MASLD), has been shown to have a positive low-risk association with pancreatic cancer in four meta-analyses, see Fig. 4. NAFLD is commonly found in obese individuals but when a fourth meta-analysis by Souza et al. explored whether the risk of NAFLD varied between lean and non-lean individuals it was found to be higher in the lean individuals (RR 1.97; 95% CI 1.01–3.86)^43^ suggesting the importance of awareness in this sub group of patients.

A large meta-analysis of 12 studies reviewed whether metabolic bariatric surgery had an effect on risk of pancreatic cancer and found that there was evidence of an association with decreased risk (RR 0.46; 95% CI 0.30–0.71).^44^

#### Pancreatitis

There is convincing evidence that the presence of a history of pancreatitis substantially increases the risk of pancreatic cancer. The highest risk is in patients with hereditary pancreatitis (RR 63.6; 95% CI 45.4–88.4).^45^ However idiopathic forms of pancreatitis are also high risk, as shown in Fig. 5, whether they be acute or chronic and of any duration since diagnosis.

#### Other

Multiple other risk factors related to a patient's comorbidities have been explored for association with pancreatic cancer and these are summarised in Fig. 5.

BRCA gene mutations have been shown in two meta-analyses to be associated with a high increased risk of pancreatic cancer (RR 2.65; 95% CI 1.43–4.91), with an indication this risk is higher in BRCA2 (RR 5.12; 95% CI 3.17–8.27) than in BRCA1 (RR 1.71; 95% CI 1.16–2.52) mutations.^46^

History of venous thromboembolism is another pre-existing condition associated with a potentially high risk of pancreatic cancer (RR 6.11; 95% CI 3.83–9.74), though this is based on only limited weak evidence from one meta-analysis.^47^

There is also evidence from four of seven meta-analyses conducted into conditions indicating poor oral health, such as periodontitis and edentulism, that they are associated with a low to moderate increased risk of pancreatic cancer. However, the most recent and largest meta-analysis or periodontitis showed no association (RR 1.35; 95% CI 1.00–1.82).^48^

Peptic ulcer disease (RR 1.26; 95% CI 1.01–1.57)^49^ and history of gastrectomy for benign disease (RR 1.54; 95% CI 1.25–1.90)^50^ were associated with an increased risk of pancreatic cancer, though each in a single meta-analysis.

Reproduction-related factors were explored across three meta-analyses and the only indication of an effect was shown for parity, with those having had a child associated with a slightly lower risk of pancreatic cancer (RR 0.91; 95% CI 0.85–0.97).^51^

Other factors shown not to be associated with pancreatic cancer were a history of non-melanoma skin cancer or cystic fibrosis.

### Measurements

#### Obesity

Many studies investigated the impact of weight on risk of pancreatic cancer as an individual risk factor or as a component of metabolic syndrome (Fig. 6).

Seven studies assessed risk of pancreatic cancer as body mass index (BMI) increased and found evidence of an association with a low increase in risk per 5 kg/m^2^ increase (RR 1.08; 95% CI 1.06–1.11).^52^

There was no difference in pancreatic cancer risk between obese individuals (defined by raised BMI or waist circumference) and those of normal weight in the most recent largest study not distinguishing by gender (RR 1.04; 95% CI 1.00–1.09). However, stratified analyses showed that females had an association with low increased risk (RR 1.17; 95% CI 1.01–1.35) whilst the association with risk for males remained non-significant (RR 1.08; 95% CI 0.85–1.37).^41^ In contrast, studies investigating the specific measurements of waist circumference and waist-hip ratio were suggestive of an association with low increased risk.^53^^,^^54^

#### Height

Increasing height has been shown in a meta-analysis of 12 studies to be associated with a low increased risk of pancreatic cancer (RR 1.07; 95% CI 1.03–1.12) (Fig. 6).^55^

#### Blood pressure

Hypertension was also shown to confer a low increased risk of pancreatic cancer (RR 1.12; 95% CI 1.08–1.17) and, similar to obesity, this was more prominent in females (RR 1.24; 95% CI 1.12–1.38) than males (RR 1.08; 95% CI 1.00–1.18) (Fig. 6).^41^

### Medication

#### Acid suppressive agents

Taking proton pump inhibitor medication is associated with a moderately increased risk of pancreatic cancer (RR 1.75; 95% CI 1.12–2.72)^56^ whilst taking the now less commonly prescribed H2 receptor antagonist medication is associated with a low increased risk (RR 1.26; 95% CI 1.02–1.57) (Fig. 6).^57^

#### Aspirin or non-steroidal anti-inflammatory drugs (NSAIDs)

Aspirin and nonsteroidal anti-inflammatory drugs have been extensively investigated and whilst there is suggestive evidence that aspirin is associated with a lowered risk of pancreatic cancer (RR 0.78; 95% CI 0.68–0.89),^58^ there is no evidence suggesting a link between other NSAIDs and pancreatic cancer (Fig. 6).

#### Diabetes medications

Ten studies examined the potential protective effect of metformin and most found a reduction in the risk of pancreatic cancer, whether compared to other antidiabetic medications (RR 0.57; 95% CI 0.35–0.93) or to no therapy (RR 0.62; 95% CI 0.45–0.84) (Fig. 7).^59^ This was the opposite to hypoglycaemic diabetes medications insulin (RR 2.85; 95% CI 1.75–4.64)^60^ and sulfonylureas (RR 1.79; 95% CI 1.29–2.49)^60^ which showed evidence of a positive association with pancreatic cancer. There was no evidence of an effect for thiazolidinediones (RR 1.13; 95% CI 0.73–1.75)^60^ or incretin-based therapies (RR 1.04; 95% CI 0.87–1.24).^61^

#### Statins

Another commonly prescribed and extensively studied class of medication is the lipid-lowering statin and there is evidence that their use is associated with a reduced risk of pancreatic cancer (RR 0.83; 95% CI 0.72–0.96) (Fig. 6).^62^

#### Renin-angiotensin-aldosterone system inhibitors

The use of angiotensin converting enzyme inhibitors or angiotensin receptor blockers, either alone or in combination with one another showed no association with a risk of pancreatic cancer.^63^

#### Hormonal therapies

The use of hormone replacement therapy (HRT) appears not to alter the risk of pancreatic cancer when all forms are taken into account (RR 0.92; 95% CI 0.83–1.02).^64^ There is evidence for a slight reduction in the risk of pancreatic cancer in people who have ever used the oral contraceptive pill compared to those who have not (RR 0.85; 95% CI 0.73–0.98) (Fig. 6).^65^

### Lifestyle factors

#### Alcohol

Six studies investigated the link between alcohol consumption and risk of pancreatic cancer but often used inconsistent classifications. In general, the only consistent positive association was a low increased risk in those drinking heavily vs. those who did not drink or drank only occasionally (RR 1.19; 95% CI 1.11–1.28) (Fig. 7).^66^

#### Smoking

The association between smoking and pancreatic cancer was explored in seven meta-analyses and universally found to be associated with increased risk, with this risk higher in current smokers (RR 1.80; 95% CI 1.70–1.90) than in those who had stopped smoking (RR 1.20; 95% CI 1.10–1.20) (Fig. 7).^67^

### Test results

#### Blood group

Having an antigen positive blood type (A, B or AB) has been shown to be associated with a higher risk of pancreatic cancer than having antigen negative blood group O (RR 1.27; 95% CI 1.11–1.43) (Fig. 8).^68^

#### Lipids

Low levels of high-density lipoprotein (HDL) cholesterol slightly increase the risk of pancreatic cancer (RR 1.24; 95% CI 1.11–1.38)^42^ but total cholesterol levels do not seem to have a significant effect (RR 0.89; 95% CI 0.79–1.00).^69^ There is mixed evidence for hypertriglyceridemia being associated with pancreatic cancer with one recent meta-analysis reporting a suggesting a low-risk association (RR 1.07; 95% CI 1.03–1.11)^41^ and the other no significant effect (RR 0.96; 95% CI 0.87–1.07) (Fig. 8).^42^

#### Liver function

Serum Gamma-Glutamyl Transferase levels were shown to have a positive association with a moderate increased risk for pancreatic cancer but only when comparing results in the highest quartile with the lowest.^70^

## Discussion

This comprehensive umbrella review of systematic reviews and meta-analyses updates the evidence on risk factors for pancreatic cancer.

This review progresses research since the 2015^10^ assessment of evidence in two important ways. First, this umbrella review has identified 58 new factors which have been investigated to assess their association with pancreatic cancer. Second, it has changed the risk categorisation of 9 of the 22 originally reported risk factors as a result of updated evidence.^10^

In the high-risk association category (RR ≥ 2.0), we confirm the association with chronic pancreatitis but show that the increased risk is across all forms of pancreatitis and all durations of diagnosis. We also confirm the findings that a history of venous thromboembolism is associated with a high risk of pancreatic cancer, but this is not restricted to idiopathic thrombosis. We newly identify patients with autoimmune liver diseases, carriers of BRCA gene mutations, patients co-infected with hepatitis B and C and those diabetics requiring insulin as at high risk of pancreatic cancer. We also suggest that family history of pancreatic cancer confers a high rather than moderate increase in risk.

We confirm that factors that show association with moderate increased risk of pancreatic cancer (RR ≥1.5–<2.0) include diabetes and smoking but recategorise metabolic syndrome to a low-risk association. We have split antidiabetic drugs into their classes and show that use of sulfonylureas is associated with a moderate increased risk of pancreatic cancer. We newly identify a further 7 risk factors that moderately increase risk: inflammatory bowel disease, non-alcoholic fatty liver disease, poor oral health, tuberculosis, being the recipient of a solid organ transplant, having a raised serum gamma-glutamyl transferase and use of proton pump inhibitor medications. We have also found evidence that increases the categorisation for hepatitis B and C and for gastrectomy for benign disease from low to moderate risk.

Of the factors we categorise as conferring a low increased risk (RR ≥1.0–<1.5), 5 factors (cholecystectomy, heavy alcohol use, increasing height and waist circumference and having the blood type O compared to A, B or AB) remain the same as in the study by Maisonneuve and Lowenfels.^10^ However, we have identified 12 new risk factors in this category: coeliac disease, gout, hypertension, non-alcoholic fatty liver disease, obstructive sleep apnoea, peptic ulcer disease, prediabetes, systemic lupus erythematosus, antibiotic use, benzodiazepine use, H2 receptor antagonist use, and the increase in risk per 5 kg/m^2^ increase in BMI.

Of the newly identified potential risk factors, 28 show no association with pancreatic cancer (i.e., the confidence interval for the RR crosses 1.0). Use of non-steroidal anti-inflammatory drugs remains non-significant but the use of aspirin specifically has been redesignated as conferring a potential reduction in risk of pancreatic cancer. Obesity has moved from a low-risk association into the category of no association, though caution must be applied here as the evidence is mixed and there is a risk that weight loss occurring as a prodrome to pancreatic cancer is obscuring the true magnitude of the association, as can be seen in the negative direction of association between obesity and pancreatic cancer in patients with new-onset diabetes.^12^ *H. pylori* infection has also moved to the category of no association from low association.

Of the factors suggesting a protective effect against pancreatic cancer (RR ≥0.5–<1.0), allergies remain important with new information on specific types of allergy that might confer protection including atopy, hay fever, respiratory and dermal allergies. Metabolic bariatric surgery is newly associated with a reduced risk of pancreatic cancer. In addition to this there is new evidence that having children may reduce the risk of pancreatic cancer. Metformin continues to show a significant amount of evidence for a protective effect. Statins and aspirin are re-classified from non-significant to protective and the oral contraceptive pill is a newly identified protective factor.

In conducting an umbrella review we have necessarily excluded individual observational studies that have not been included in systematic reviews or meta-analyses and therefore may have missed newly investigated risk factors or those with low prevalence that may not yet have enough evidence to have a systematic review or meta-analysis performed. However, we did identify a large number of potential factors and on discussion between clinical colleagues there was no suggestion that any known factors had been missed. We will also have included results from original studies more than once where there are multiple meta-analyses on the same topic. We have addressed this by reporting all the results in our detailed summary table and in the figures summarising all the results but ensuring the ultimate rating of the risk was predicated on the results of a single meta-analysis. We purposefully avoided combining meta-analysis results either by further meta-analysis or other statistical technique to avoid giving individual component studies undue weighting.

Our credibility assessment score was important for giving a sense of the level of evidence but the lack of data on numbers of patients with pancreatic cancer included in each study limited its efficacy. Case numbers were only available for 230 of the 365 extracted meta-analyses and for those 135 meta-analyses without information on case numbers, the highest credibility rating their evidence could achieve was ‘weak’ as they automatically failed on the criteria of having >1000 cases.

Our review was also limited by only using those meta-analyses and systematic reviews published in English language but the inclusion of studies that analysed studies in all languages to start with negated this to some extent as the original non-English language studies could be included through those studies.

There was a risk of introducing selection bias in our results due to many of the original included reviews being based on results of electronic health records. This could lead to underrepresentation of asymptomatic individuals or those with limited access to healthcare, although the results remain relevant to the consulting population. This was somewhat mitigated by the inclusion of population-based cohort studies in many of the reviews but this potential for selection bias should be taken into consideration when interpreting the results of this umbrella review.

The decision not to include environmental factors and the majority of genomic factors in our review means that there are known risk factors for pancreatic cancer missing from our results. Our conclusions must therefore be taken in the context of other reviews into the association of these specific factors with pancreatic cancer.

A final limitation was the potential confounding of results by the crossover between features of an undiagnosed pancreatic cancer, e.g., new-onset diabetes or weight loss, and the risk factors that may have increased an individual's prior risk of developing the disease, e.g., diabetes and obesity. We did not address this specifically in this review but did extract information on temporality of the diagnosis of diabetes and included the results of a review of patients with new-onset diabetes and other risk factors including obesity.^12^ This will be an important area for a more focussed review in future.

The impact of this umbrella review can be best understood in terms of why the identification of risk factors is useful. The risk factors identified are particularly useful for three key reasons: targeting prevention and surveillance, developing risk prediction models and directing future research.

In terms of targeting prevention and surveillance, there are three main uses of the collated knowledge of risk factors. Firstly, any risk factors that are modifiable, e.g., smoking, can be targeted for prevention of cancer through interventions aimed at reducing their impact. Secondly, populations with non-modifiable risk factors, such as autoimmune diseases, can be targeted in future prevention strategies aimed at reducing their risk to the population baseline through stringent modification of modifiable risk factors. Finally, in the new landscape of surveillance using tools such as MCEDs, it will be important to know which patients have an increased prior risk of pancreatic cancer.^6^ These patients can then be targeted both in trials and real-world usage of the tools.

Risk prediction models (for both prediction of undiagnosed cancer risk in symptomatic patients and prediction of future cancer risk to inform surveillance and prevention strategies) need a comprehensive set of risk factors to serve as candidate variables for their development. In the majority of cases at present there is a lack of evidence underpinning the identification and selection of these risk factors.^71^ Not using a systematic approach to identifying risk factors can result in important factors being missed and thus models not performing optimally.^72^ The solution to this could be the implementation of systematic approaches such as the one described in this umbrella review.

A final important use of the results of this umbrella review is informing future research in the area. Having the evidence on risk factors available at a glance can help inform future prioritisation of studies and reviews, for example by concentrating resources on those risk factors which are less well studied or have contradictory evidence. For those risk factors with significant evidence already, the focus could shift towards understanding if there is a causal association and any potential mechanisms.

## Contributors

SM and GA designed the study and developed the search strategy. SM, SP, JK, and SB performed the systematic review and data extraction. SM curated the data and analysed it. GA and SP had full access to and verified the underlying data. SM and GA developed the visualisation of the data. First draft writing was performed by SM and all authors contributed to review and editing. All authors approved the final manuscript. Funding was acquired by SM.

## Data sharing statement

No new data was generated during this study. All data extracted for the umbrella review is made available in detail in Appendix 1. Any further details are contained in the original publications, the references for which are contained either in the paper or in Appendix 1. The protocol for the study has already been published and contained a statistical plan.

## Declaration of interests

SM declares a doctoral fellowship for primary care clinicians awarded from Wellcome, grant number PMHG1A4. RN declares grants or contracts from Cancer Research UK, the National Institute for Health Research (the views expressed are those of the authors and not necessarily those of the NIHR or the Department of Health and Social Care), Innovate UK, Cancer Research Wales and Yorkshire Cancer Research. RN declares he is a named inventor of the PinPoint Test and if it generates revenue in the future then he may receive a revenue share. RN declares consulting fees from GRAIL as co-chief investigator on the NHS Galleri trial. RN declares unpaid trustee roles on Prostate Cancer UK and the Southwest GP Trust. All other authors declare no interests.
